# Supplementary material for: Whole Transcriptome Analyses of Apricots and Japanese Plum Fruits after 1-MCP (Ethylene-Inhibitor) and Ethrel (Ethylene-Precursor) Treatments Reveal New Insights into the Physiology of the Ripening Process
Source: Int J Mol Sci. 2022 Sep 20;23(19):11045. doi: 10.3390/ijms231911045 (PMC9569840; doi:10.3390/ijms231911045)
Supplement: Supplementary file 1 [file ijms-23-11045-s001.zip › Supplementary Tables/Suplementary Tables (S1 to S4).pdf]

Table S1. Raw data stats for apricot ‘Goldrich’ (GD) and Japanese plum ‘Santa Rosa’ (SNR) including a control (T0) and two treatments (1-MCP, T1 and ethrel, T2) as well as three replicates per treatment (A, B and C).

Sample ID	Total read bases	Total reads	GC (%)	Q20	Q30
GD-T0-A	9,408,660,812	62,309,012	46.1	96.93	91.78
GD-T0-B	9,566,073,480	63,351,480	45.96	97.17	92.21
GD-T0-C	8,931,888,580	59,151,580	46.22	97.13	92.09
GD-T1-A	9,242,216,834	61,206,734	45.95	97.15	92.17
GD-T1-B	8,645,798,846	57,256,946	46.66	97.07	92.23
GD-T1-C	8,616,004,734	57,059,634	45.90	96.81	91.42
GD-T2-A	9,749,954,636	64,569,236	45.47	97.10	92.03
GD-T2-B	9,082,979,784	60,152,184	45.99	97.00	91.92
GD-T2-C	7,225,079,408	47,848,208	45.88	96.84	91.48
SNR-T0-A	7,150,458,832	47,354,032	46.39	96.95	91.72
SNR-T0-B	7,169,963,502	47,483,202	45.89	96.93	91.68
SNR-T0-C	8,521,170,996	56,431,596	45.81	97.11	92.04
SNR-T1-A	7,343,752,120	48,634,120	46.19	96.63	91.11
SNR-T1-B	7,903,112,292	52,338,492	46.72	96.63	91.07
SNR-T1-C	8,869,491,454	58,738,354	46.27	96.76	91.31
SNR-T2-A	9,702,466,646	64,254,746	46.4	97.41	92.71
SNR-T2-B	10,276,368,420	68,055,420	46.18	97.23	92.27
SNR-T2-C	9,344,111,332	61,881,532	46.27	97.24	92.31

Total read bases: Total number of bases sequenced
Total reads: Total number of reads
GC (%): GC content
Q20 (%): Ratio of bases that have phred quality score greater than or equal to 20
Q30 (%): Ratio of bases that have phred quality score greater than or equal to 30

Table S2. Trimming data stats for apricot ‘Goldrich’ (GD) and Japanese plum ‘Santa Rosa’ (SNR) including a control (T0) and two treatments (1-MCP, T1 and ethrel, T2) as well as three replicates per treatment (A, B and C).

Sample ID	Total read bases	Total reads	GC(%)	Q20	Q30
GD-T0-A	8,876,484,974	61,004,076	45.99	97.79	93.05
GD-T0-B	9,127,950,897	62,288,240	45.91	97.89	93.28
GD-T0-C	8,509,019,717	58,134,278	46.13	97.84	93.15
GD-T1-A	8,822,723,860	60,124,492	45.90	97.87	93.24
GD-T1-B	8,026,138,929	56,114,096	46.47	98.03	93.62
GD-T1-C	8,217,861,834	55,994,724	45.84	97.59	92.56
GD-T2-A	9,339,043,173	63,428,126	45.42	97.81	93.07
GD-T2-B	8,669,361,723	58,926,992	45.95	97.81	93.09
GD-T2-C	6,893,666,152	46,924,412	45.83	97.60	92.61
SNR-T0-A	6,826,470,226	46,483,942	46.35	97.69	92.82
SNR-T0-B	6,848,033,928	46,593,278	45.84	97.69	92.80
SNR-T0-C	8,148,738,444	55,446,922	45.73	97.82	93.10
SNR-T1-A	6,953,085,901	47,617,922	46.12	97.52	92.43
SNR-T1-B	7,520,750,017	51,243,158	46.69	97.49	92.32
SNR-T1-C	8,444,387,459	57,553,810	46.24	97.56	92.48
SNR-T2-A	9,249,882,757	63,360,424	46.30	98.04	93.65
SNR-T2-B	9,852,936,763	67,065,126	46.11	97.87	93.21
SNR-T2-C	8,941,129,524	60,984,118	46.18	97.87	93.24

Total read bases: Total number of bases sequenced
Total reads: Total number of reads
GC (%): GC content
Q20 (%): Ratio of bases that have phred quality score greater than or equal to 20
Q30 (%): Ratio of bases that have phred quality score greater than or equal to 30

Table S3. *De novo* Transcriptome assembly statistics for apricot ‘Goldrich’

Assembly	All transcript contigs	Only longest isoform per 'gene'
Total trinity 'genes'	81,894	81,894
Total trinity transcripts	102,718	81,894
GC (%)	41.47	41.45
N90	308	280
N80	477	406
N70	710	591
N60	1,003	855
N50	1,336	1,204
N40	1,680	1,589
N30	2,057	1,994
N20	2,529	2,487
N10	3,298	3,274
Maximum contig length	13,689	13,689
Minimum contig length	201	201
Median contig length	447,0	393.0
Average contig length	788,60	715,20
Total assembled bases	81,003,397	58,570,553

-Total trinity genes: Total number of assembled genes by Trinity
-Total trinity transcripts: Total number of assembled transcripts by Trinity
-Percent GC: GC percent
-N[x] length statistic: at least x% of the assembled transcript nucleotides are found in contigs that are at least of Nx length. The traditional method is computing N50, such that at least half of all assembled bases are in transcript contigs of at least the N50 length value. The contig N50 values can often be exaggerated due to an assembly program generating too many transcript isoforms, especially for the longer transcripts. To mitigate this effect, Nx values are also calculated based on the longest isoform per gene.
-Maxium/Minimum/Median/Average contig length: Maxium/Minimum/Median/Averaged length of assembled transcripts
-Total assembled bases: The left column is total bases of assembled contigs and right column is total bases of the longest isoform.

Table S4. *De novo* Transcriptome assembly statistics for Japanese plum 'Santa Rosa'

Assembly	ALL transcript contigs	Only longest isoform per 'gene'
Total trinity 'genes'	74,999	74,999
Total trinity transcripts	96,864	74,999
GC (%)	41,39	41,37
N90	317	291
N80	481	416
N70	712	600
N60	1,015	885
N50	1,348	1,256
N40	1,694	1,641
N30	2,054	2,033
N20	2,504	2,494
N10	3,279	3,269
Maximum contig length	14,450	14,450
Minimum contig length	201	201
Median contig length	456	404
Average contig length	798.77	732.73
Total assembled bases	77,371,727	54,953,688

-Total trinity genes: Total number of assembled genes by Trinity
-Total trinity transcripts: Total number of assembled transcripts by Trinity
-Percent GC: GC percent
-N[x] length statistic: at least x% of the assembled transcript nucleotides are found in contigs that are at least of Nx length. The traditional method is computing N50, such that at least half of all assembled bases are in transcript contigs of at least the N50 length value. The contig N50 values can often be exaggerated due to an assembly program generating too many transcript isoforms, especially for the longer transcripts. To mitigate this effect, Nx values are also calculated based on the longest isoform per gene.
-Maxium/Minimum/Median/Average contig length: Maxium/Minimum/Median/Averaged length of assembled transcripts
-Total assembled bases: The left column is total bases of assembled contigs and right column is total bases of the longest iso
