# Supplementary figures and images for: Whole Transcriptome Analyses of Apricots and Japanese Plum Fruits after 1-MCP (Ethylene-Inhibitor) and Ethrel (Ethylene-Precursor) Treatments Reveal New Insights into the Physiology of the Ripening Process
Source: Int J Mol Sci. 2022 Sep 20;23(19):11045. doi: 10.3390/ijms231911045 (PMC9569840; doi:10.3390/ijms231911045)

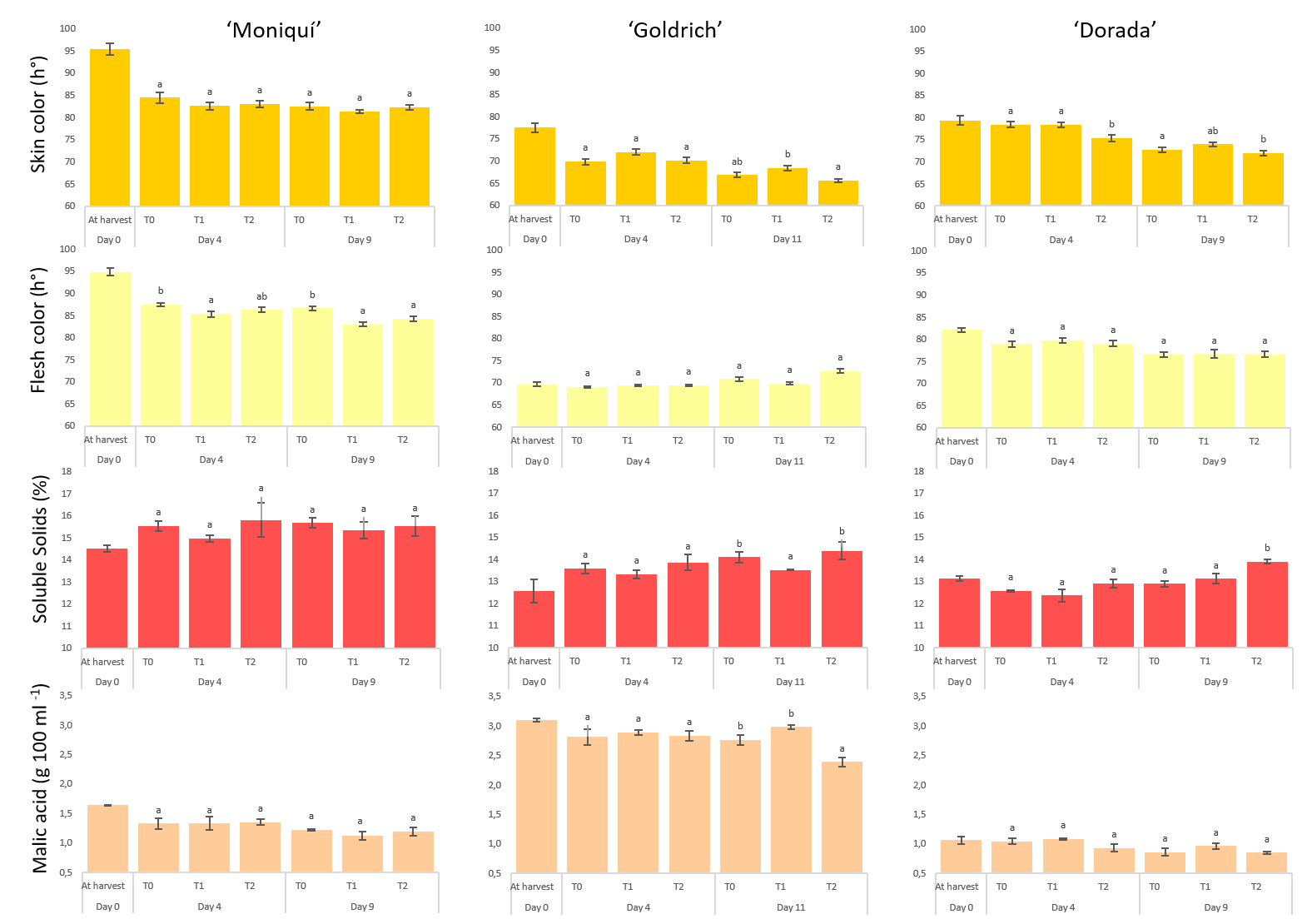

Supplement: Supplementary file 1 [file ijms-23-11045-s001.zip › Supplementary Figures/Fig. S1.png]

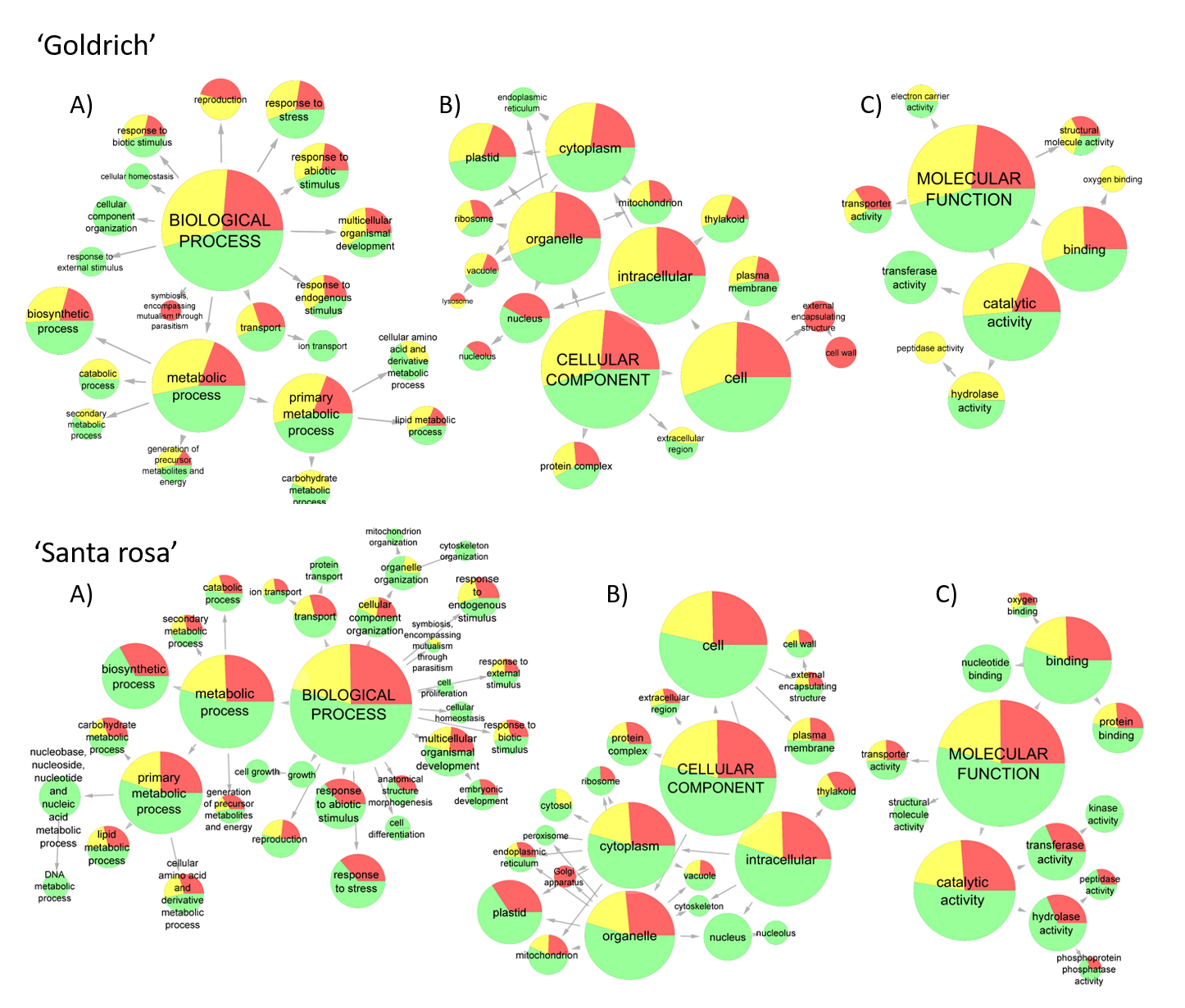

Supplement: Supplementary file 1 [file ijms-23-11045-s001.zip › Supplementary Figures/Fig. S10.png]

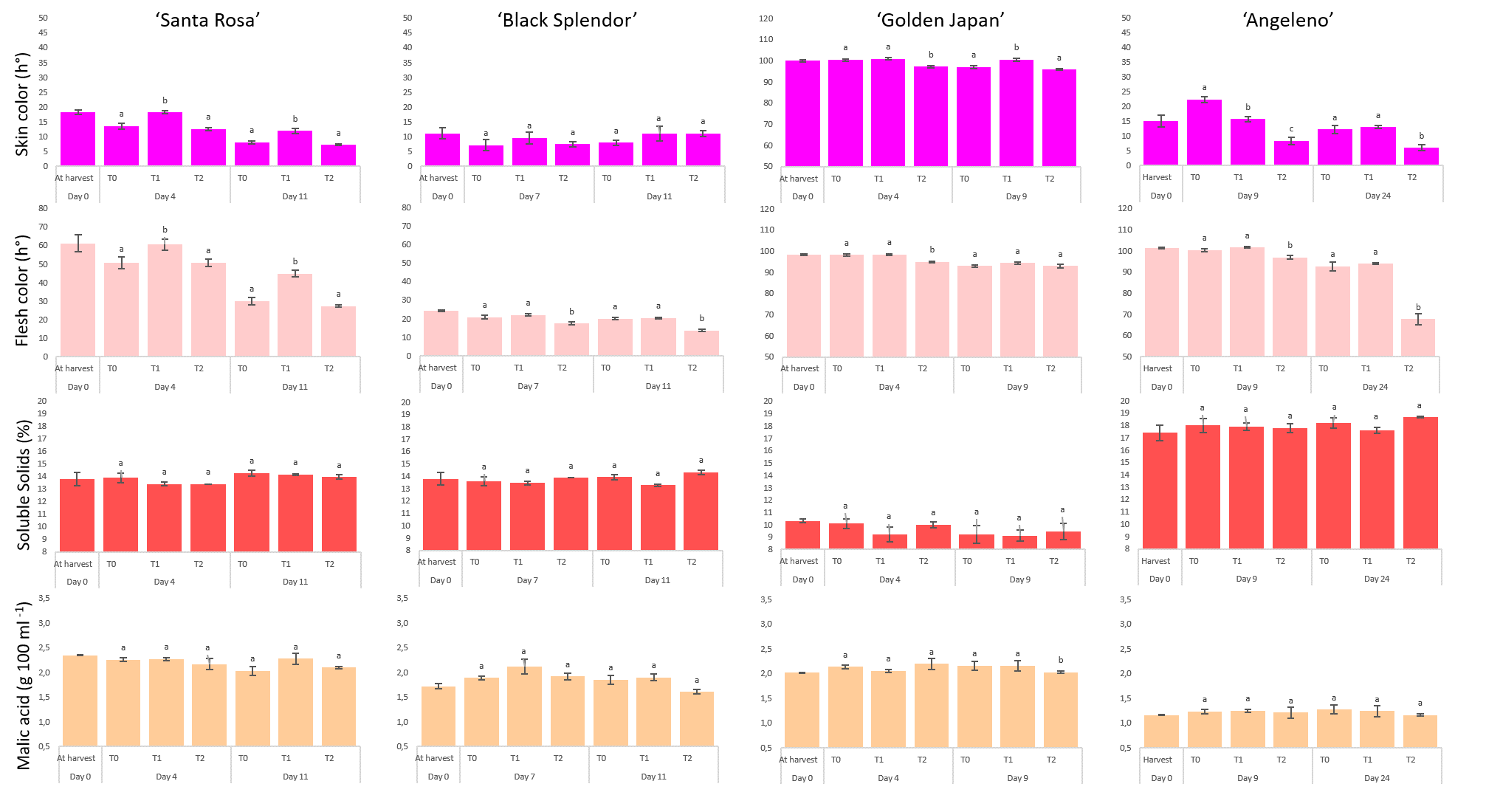

Supplement: Supplementary file 1 [file ijms-23-11045-s001.zip › Supplementary Figures/Fig. S2.png]

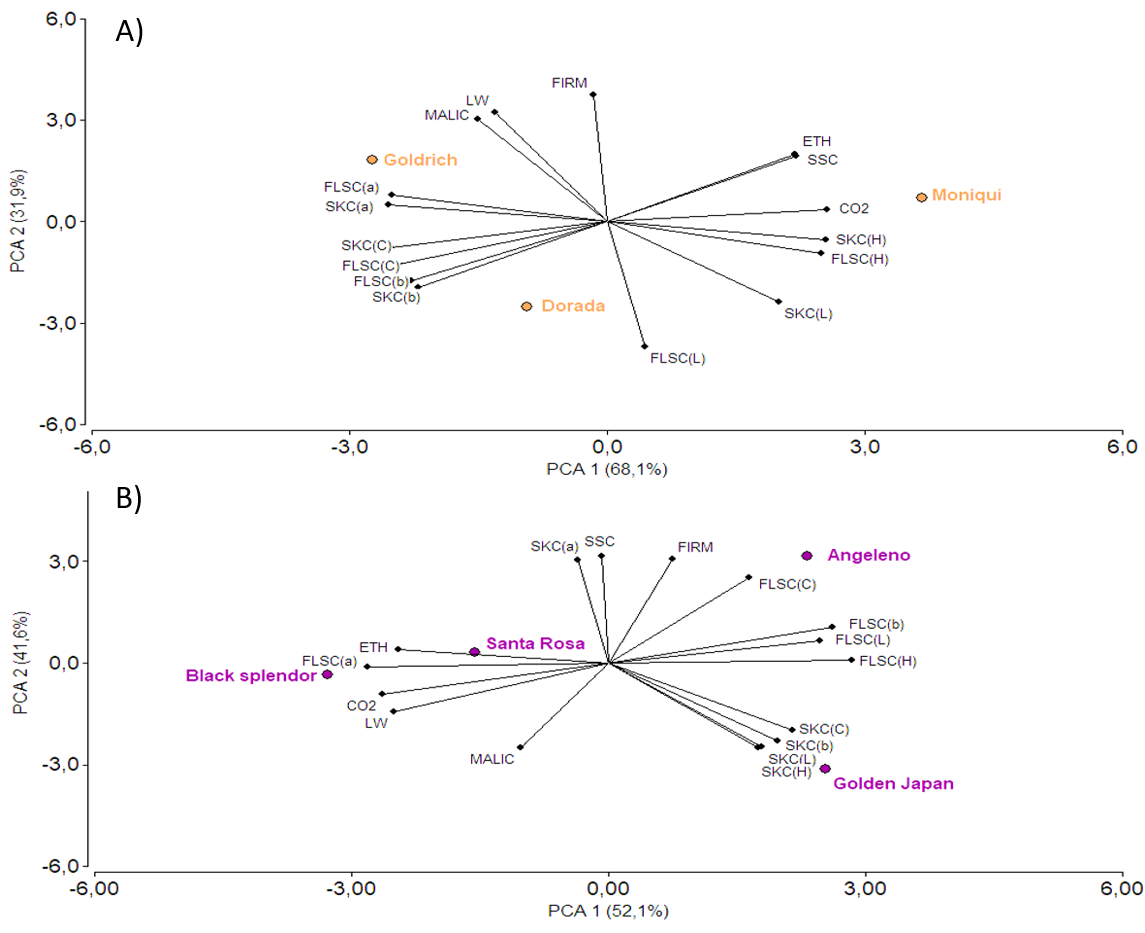

Supplement: Supplementary file 1 [file ijms-23-11045-s001.zip › Supplementary Figures/Fig. S3.png]

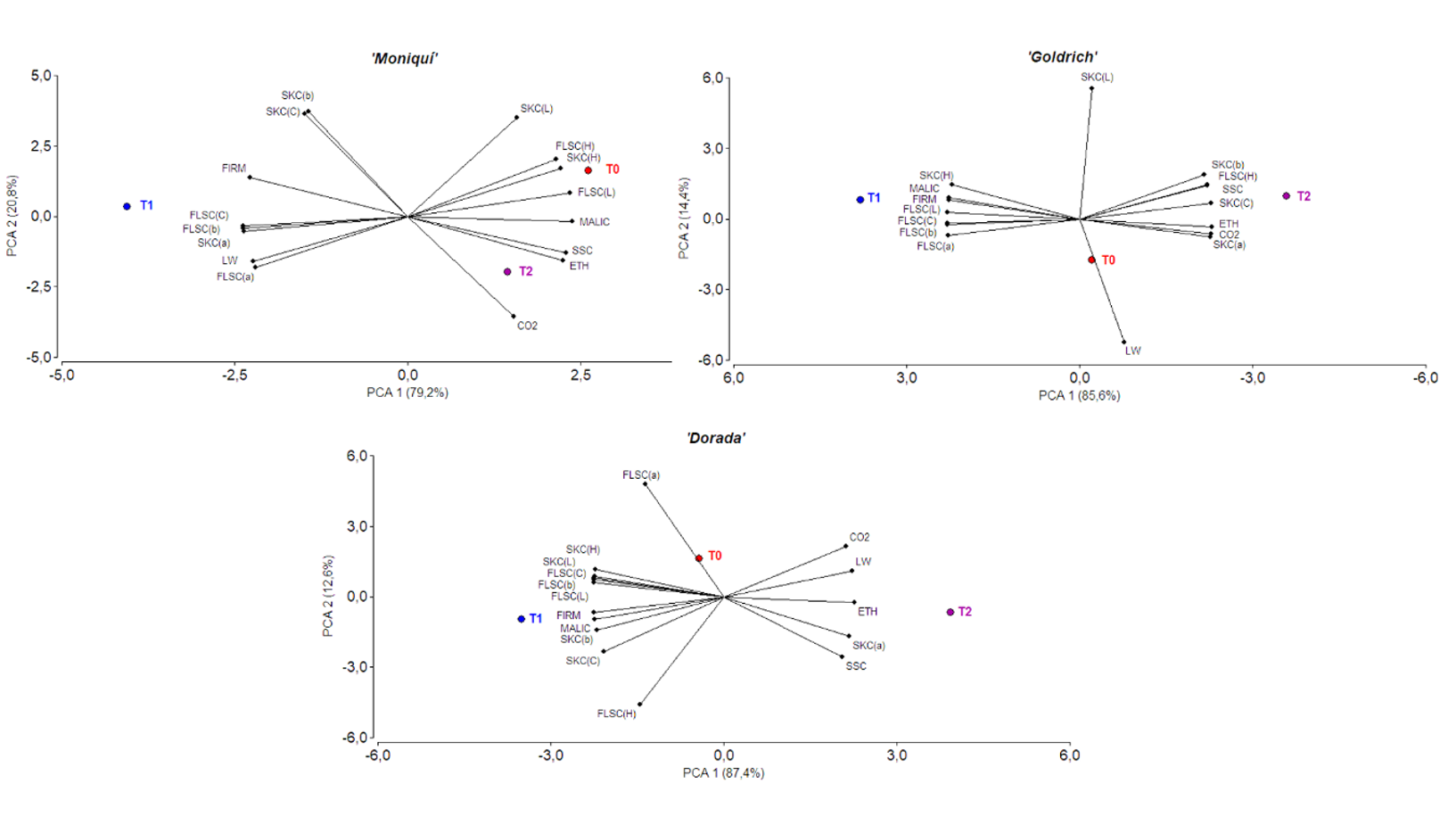

Supplement: Supplementary file 1 [file ijms-23-11045-s001.zip › Supplementary Figures/Fig. S4.png]

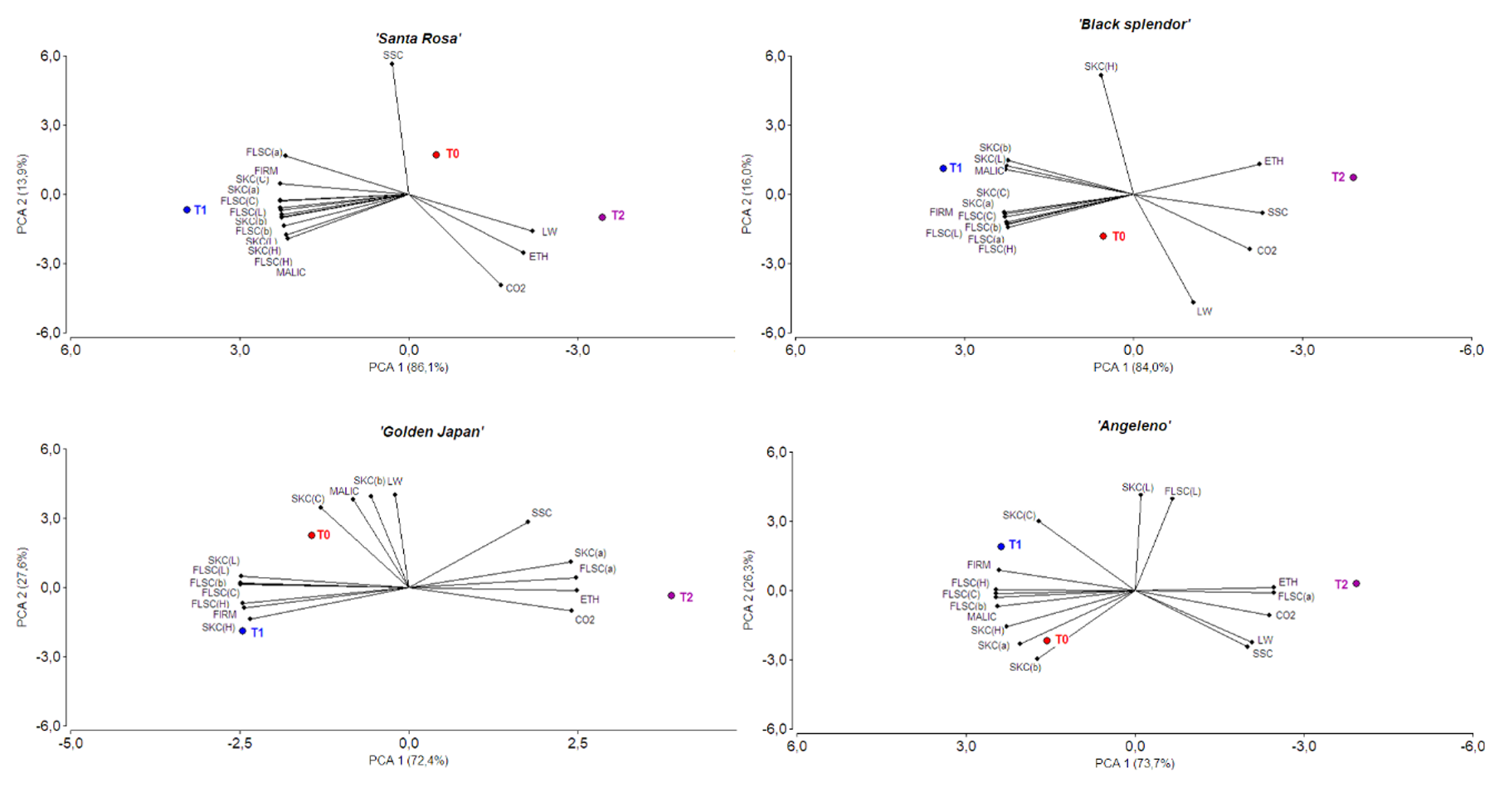

Supplement: Supplementary file 1 [file ijms-23-11045-s001.zip › Supplementary Figures/Fig. S5.png]

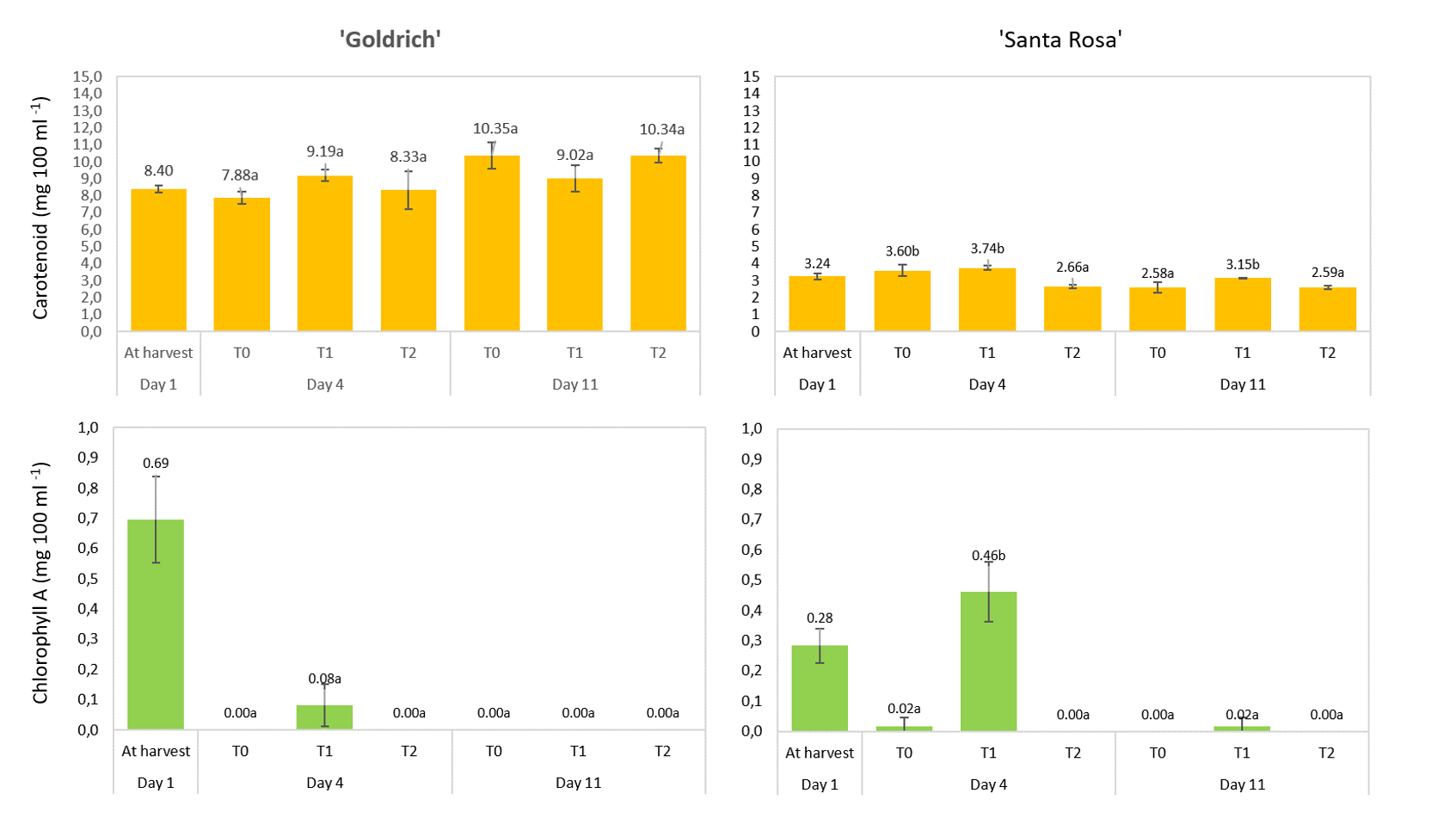

Supplement: Supplementary file 1 [file ijms-23-11045-s001.zip › Supplementary Figures/Fig. S6.png]

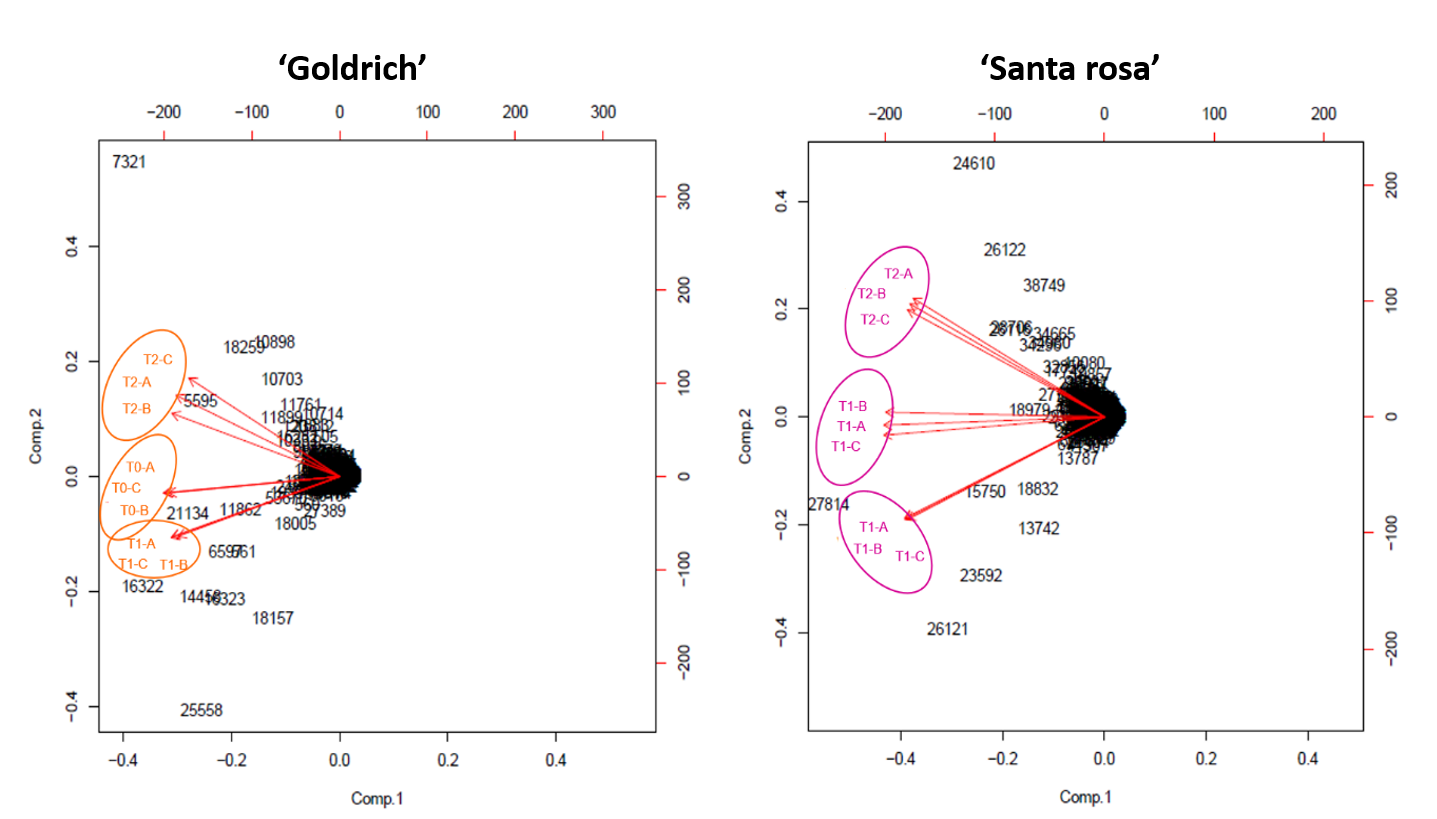

Supplement: Supplementary file 1 [file ijms-23-11045-s001.zip › Supplementary Figures/Fig. S7.png]

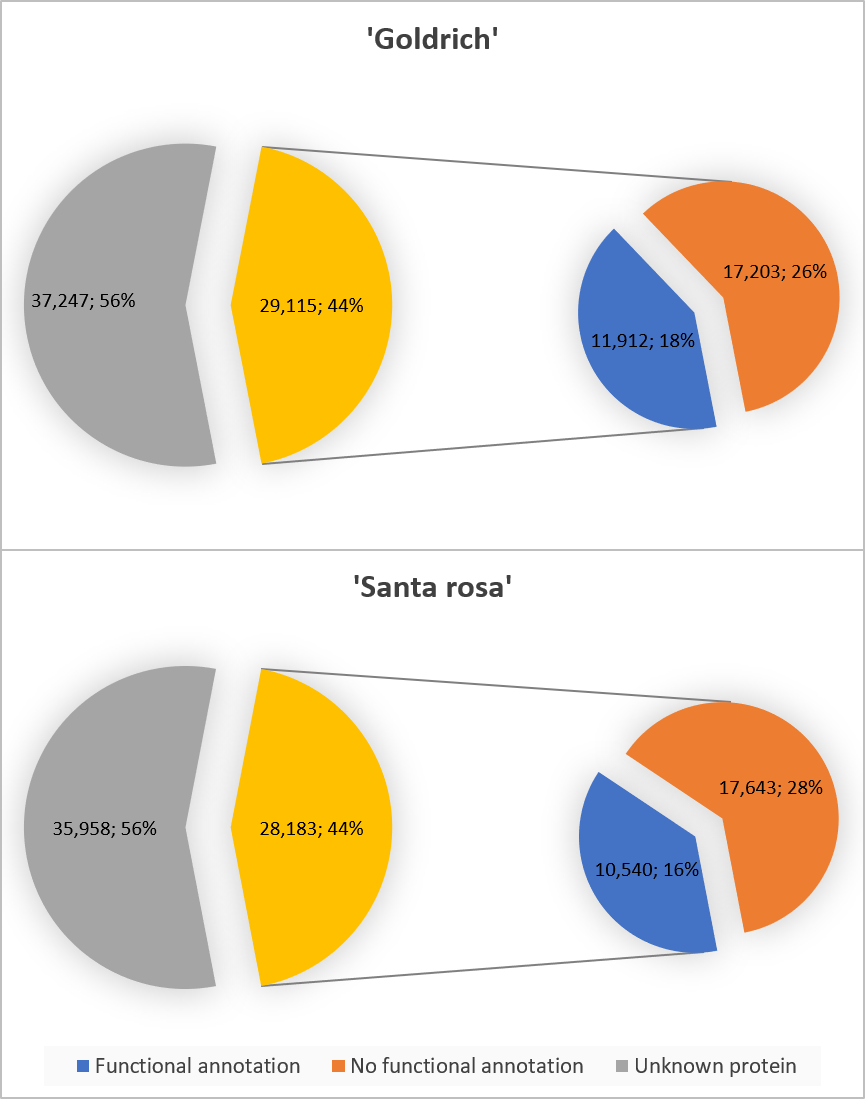

Supplement: Supplementary file 1 [file ijms-23-11045-s001.zip › Supplementary Figures/Fig. S8.png]

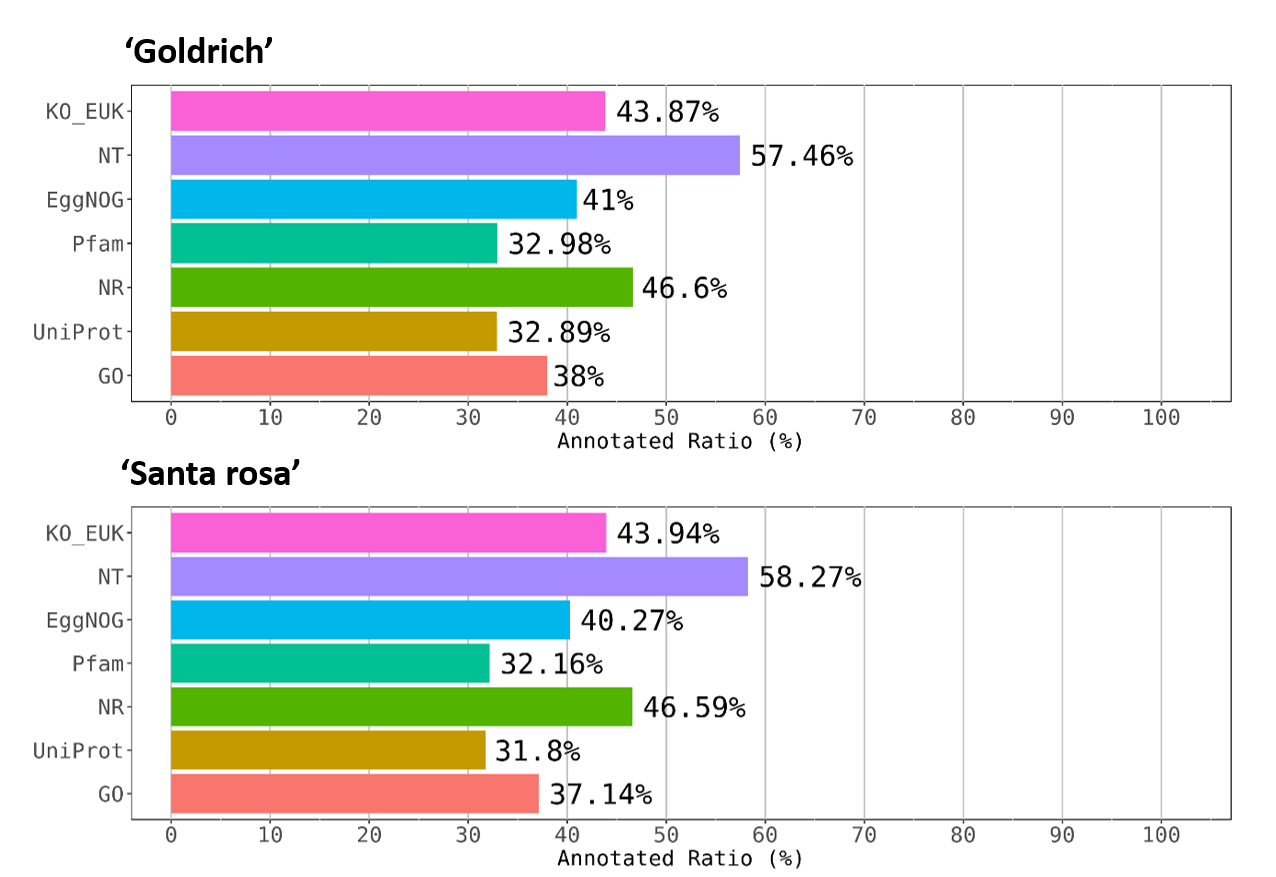

Supplement: Supplementary file 1 [file ijms-23-11045-s001.zip › Supplementary Figures/Fig. S9.png]
